# Leveraging imperfection with MEDLEY: a multi-model approach harnessing bias in medical AI

**DOI:** 10.3389/frai.2026.1701665

**Published:** 2026-03-04

**Authors:** Farhad Abtahi, Mehdi Astaraki, Fernando Seoane

**Affiliations:** 1 Department of Clinical Science, Intervention and Technology, Karolinska Institutet, Stockholm, Sweden; 2 Department of Biomedical Engineering and Health System, School of Engineering Sciences in Chemistry, Biotechnology and Health, KTH Royal Institute of Technology, Huddinge, Sweden; 3 Department of Clinical Physiology, Karolinska University Hospital, Stockholm, Sweden; 4 Department of Textile Technology, Faculty of Textiles, Engineering and Business Swedish School of Textiles, University of Borås, Borås, Sweden; 5 Department of Medical Technologies, Karolinska University Hospital, Huddinge, Sweden

**Keywords:** AI regulation and governance, bias and fairness in AI, clinical decision support systems, diagnostic uncertainty, hallucination in large language models, human-in-the-loop AI, medical artificial intelligence, multi-model and ensemble learning

## Abstract

Bias in medical artificial intelligence is conventionally viewed as a defect that requires elimination. However, human reasoning inherently incorporates biases shaped by education, culture, and experience, suggesting their presence may be inevitable and potentially valuable. We propose MEDLEY (Medical Ensemble Diagnostic system with Leveraged diversitY), a conceptual framework that orchestrates multiple AI models while preserving their diverse outputs rather than collapsing them into a consensus. Unlike traditional approaches that suppress disagreement, MEDLEY documents model-specific biases as potential strengths and treats hallucinations as provisional hypotheses for clinician verification. A proof-of-concept demonstrator for differential diagnosis was developed using over 30 large language models, preserving both consensus and minority views, rendering diagnostic uncertainty and latent biases transparent to support clinical oversight. While not yet a validated clinical tool, the demonstration illustrates how structured diversity can enhance medical reasoning under the supervision of clinicians. By reframing AI imperfection as a resource, MEDLEY offers a paradigm shift that opens new regulatory, ethical, and innovation pathways for developing trustworthy medical AI systems.

## 1 Introduction

In the seven decades since Artificial Intelligence (AI) was first defined at Dartmouth, we have witnessed a rapid rise in attention over the past few years, mainly driven by public access to large language models (LLMs) and the push to integrate them into nearly every aspect of daily life. AI has rapidly expanded into medicine, with over 1,000 FDA-cleared tools already available for use across various domains, including imaging, diagnostics, and workflow support. LLMs have accelerated expectations for clinical integration, offering capabilities such as summarizing patient records, generating reports, and assisting in diagnosis and treatment planning. However, despite their fame, no LLM-based system has been reviewed or approved by the FDA for clinical use (Warraich et al., 2025). This gap reflects both the promise of these models and the unresolved risks they pose.

Bias, systematically favoring or disadvantaging certain groups, remains a pervasive challenge (Gianfrancesco et al., 2018; Mehrabi et al., 2021). Hallucinations, where models generate ungrounded outputs, are especially concerning in clinical contexts (Ji et al., 2023). Moreover, the “black-box” nature of deep learning systems complicates accountability and trust (Sendak et al., 2020). These limitations, i.e., bias, hallucination, and opacity, are well-documented in the biomedical and computer science literature, highlighting the ethical stakes of deploying AI in high-risk domains such as healthcare (Cabitza et al., 2017; Chen et al., 2021).

Regulators are responding with layered frameworks. The FDA emphasizes a lifecycle, risk-based approach to AI oversight, while the EU’s Medical Device Regulation (MDR), *In Vitro* Diagnostic Regulation (IVDR), and AI Act designate medical AI systems, especially those used for diagnosis or treatment, as “high-risk,” requiring bias mitigation, transparency, and human oversight. The EU has further advanced this by developing structured AI evidence pathways to operationalize trustworthy AI in healthcare, a roadmap for demonstrating safety, efficacy, and accountability across an AI system’s lifecycle (Griesinger et al., 2025). These guidelines aim to standardize documentation and validation evidence, ensure robust model performance across diverse populations, and support traceability, evaluation, and post-market monitoring.

These concerns echo a deeper question of responsibility: when an AI system fails, who is accountable? Clinicians often feel they remain ultimately liable, even when decisions are influenced by opaque AI systems (Nouis et al., 2025). Philosophical analyses describe this as a “responsibility gap,” where neither clinicians, developers, nor institutions can be blamed for AI-driven harm (Barman et al., 2025). The current consensus holds that AI models must function as decision-support systems, with the final decision remaining in the hands of the human expert. However, for this responsibility to be meaningful, clinicians must understand the basis of AI outputs. Explainable AI has been proposed as one solution. However, research shows that when explanations are overly persuasive, they can exacerbate automation bias, encouraging clinicians to overtrust outputs even when they are incorrect (Romeo and Conti, 2025). Similarly, recent analyses of large language models reveal that their behavior can echo human cognitive shortcuts, similar to Kahneman’s “System 1” fast thinking, where seemingly intuitive but shallow reasoning ^1^can lead to hallucinations or overconfident errors (Hagendorff et al., 2023). Together, these insights highlight the limitations of relying solely on explanation and the need for frameworks that preserve the diversity of perspectives and explicitly surface uncertainty.

This study introduces MEDLEY (Medical Ensemble Diagnostic system with LEveraged diversitY) as an abstract framework for bias-aware and hallucination-tolerant medical AI. MEDLEY is a conceptual paradigm that treats bias and potentially hallucination as structured resources for decision support. Unlike traditional ensembles that collapse multiple outputs into a single answer, MEDLEY emphasizes diversity, transparency, plurality, and context by orchestrating various models in parallel. This conceptual framework applies to different domains; however, a proof-of-concept system was developed to demonstrate its feasibility. This demonstrator utilized over 30 LLMs across clinical cases to investigate diagnostic variability, bias attribution, and overall system performance. We then discuss how MEDLEY, as a conceptual approach, has broader implications for explainability, ethics, regulation, and innovation in medical AI.

## 2 Method

The MEDLEY framework is guided by four principles: *diversity*, *transparency*, *plurality*, and *context*. Unlike traditional ensemble methods that aggregate outputs into a single prediction, MEDLEY deliberately preserves multiple model outputs and their bias profiles to enrich clinical reasoning. *Diversity* ensures models with heterogeneous training protocols and/or learning algorithm architectures are included. *Transparency* requires documentation of provenance and limitations. *Plurality* emphasizes preserving distinct outputs rather than resolving conflicting predictions by highlighting the consensus. *Context* enables clinicians to interpret results relative to patient-specific factors (Amann et al., 2020; Suresh and Guttag, 2021). Together, these principles reframe bias and hallucination not as defects but as resources for structured decision support.

Ensemble learning has historically been framed in terms of error reduction. Classical methods, such as bagging and boosting, aggregate model outputs to suppress variance, while Bayesian ensembles integrate predictions into a single posterior-weighted distribution. Mixture-of-experts frameworks go further, using a gating network to route inputs to the “most appropriate” expert, collapsing plurality into an optimized pathway (Dietterich, 2000). In all these paradigms, disagreement is treated as noise, and bias is managed implicitly through weighting or selection. While highly effective for benchmark accuracy, these approaches obscure the diversity of perspectives that may carry clinical significance. Table 1 contrasts classical ensemble paradigms with MEDLEY’s approach.

**Table 1:** Comparative paradigms in ensemble learning.

Aspect	Traditional ensemble (bagging/boosting)	Bayesian ensemble	Mixture-of-experts	MEDLEY paradigm
Aim	Maximize predictive accuracy by collapsing outputs into a single result	Compute the weighted posterior average across models	Route input to the most relevant “expert” via a gating network	Provide multiple diverse perspectives for clinician synthesis
Output	Single aggregated prediction (e.g., voting and weighted sum)	Probabilistic single prediction with posterior weighting	Single optimized output from selected expert(s)	Multiple outputs with provenance and bias annotations
Treatment of disagreement	Treated as noise/variance to be minimized	Down-weights outlier models	Suppressed by gating	Preserved as a structured signal of diagnostic uncertainty
Bias handling	Attempt to cancel/eliminate bias statistically	Treated as a prior or likelihood artifact	Hidden within gating decisions	Document, contextualize, and leverage bias transparently
Hallucinations/errors	Suppressed as an error	Integrated probabilistically	Filtered via gating	Used as speculative hypotheses with human verification
Clinician cognition	Risk of automation bias; anchoring on a single output	May obscure minority perspectives under the averaged posterior	Reinforces the dominant pathway chosen by the gate	Encourages deliberation; surfaces disagreement patterns
Analogy	-Averaged poll collapsing individual votes into a single result -Single symphony performance with unified interpretation	-Weighted belief distribution -Single symphony performance	-Referral to a single specialist -Single symphony performance	-Consultation panel preserving each expert’s distinct perspective -Musical medley preserving distinct voices and styles

Classical ensembles (bagging/boosting), Bayesian ensembles, and mixture-of-experts aim to maximize predictive accuracy by collapsing multiple models into a single optimized output. In contrast, MEDLEY reframes ensemble learning for clinical contexts: disagreement is preserved as a marker of diagnostic uncertainty, and bias is treated as transparent specialization rather than error to be eliminated. This shift moves optimization away from accuracy alone toward structured diversity, provenance, and interpretability for human-in-the-loop decision making.

MEDLEY diverges from these traditional approaches by preserving rather than collapsing disagreement and optimizing for diagnostic diversity rather than aggregate accuracy. This conceptual shift reframes ensemble learning for high-stakes clinical contexts, where visibility of uncertainty, population-specific knowledge, and plural perspectives may be more valuable than marginal gains in predictive accuracy.

### 2.1 Bias taxonomy

**Figure 1:**
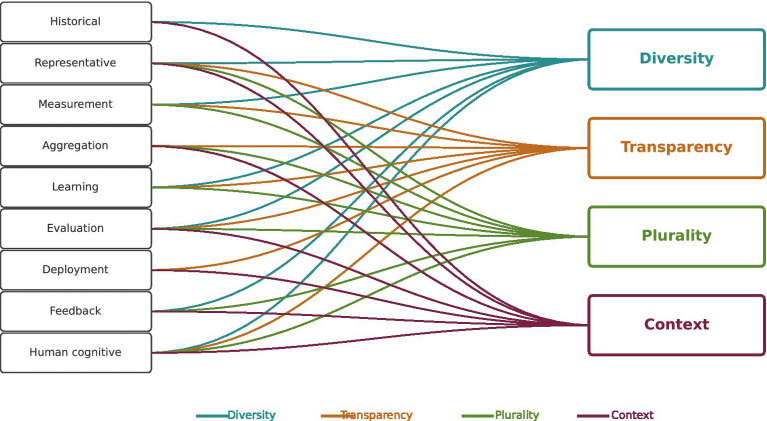
Bias categories and their alignment with MEDLEY principles. Nine types of bias common in medical AI are mapped against MEDLEY’s four guiding principles. Rather than eliminating bias, MEDLEY treats bias as specialization when transparently documented, allowing context-sensitive use of diverse model perspectives.

Bias in medical AI arises from multiple sources, including historical, demographic, measurement, and deployment-related factors. Such biases are pervasive in systems trained on electronic health records (EHRs) and are well-documented across the ML lifecycle (Suresh and Guttag, 2021). Rather than treating bias solely as a liability, MEDLEY interprets it as a form of specialization that can provide clinically relevant insight. For example, geographic biases may improve recognition of regional diseases, while temporal biases capture evolving diagnostic practices. By explicitly documenting provenance and maintaining model plurality, MEDLEY allows clinicians to weigh outputs with awareness of their limitations. Table 2 summarizes nine categories of bias and the orchestration strategies MEDLEY applies, while Figure 1 visualizes their alignment with the four guiding principles (Chen et al., 2021).

**Table 2:** Taxonomy of bias in clinical AI and MEDLEY’s orchestration strategies.

Bias category	Definition	Clinical example	MEDLEY response
Historical	Outdated concepts embedded in data	Old diagnostic criteria miss recent discoveries	MEDLEY applies the principle of diversity by incorporating models trained on temporally distinct cohorts and the principle of transparency by leveraging documented temporal provenance, thereby capturing the evolution of medical knowledge.
Representation	Training-population mismatch	Western-heavy datasets; race/gender imbalance	MEDLEY enacts diversity by selecting models trained on demographically and geographically varied populations and plurality by preserving subgroup-specific outputs that reflect meaningful variation in disease prevalence.
Measurement	Proxy variables oversimplify or misrepresent	ZIP code as genetic proxy; race coded as biology; inconsistent annotation practices	MEDLEY advances transparency by exposing proxy definitions and context by allowing clinicians to interpret model outputs in light of differing measurement conventions and their appropriateness for individual patients.
Aggregation	Loss of subgroup nuance	Pooling pediatrics/adult data or male/female physiology	MEDLEY promotes plurality by retaining subgroup-specific models and context by enabling clinicians to weight such models according to patient characteristics.
Learning	Algorithmic choices amplify disparities	Majority-class overweighting; underfitting rare subgroups	MEDLEY leverages diversity by integrating models with heterogeneous architectures and inductive biases, and plurality by preserving their distinct outputs rather than collapsing them.
Evaluation	Test data unrepresentative	Benchmarks omit rare diseases; validation lacks subgroup diversity	MEDLEY applies diversity by incorporating models validated across heterogeneous benchmarks and transparency by reporting subgroup-specific performance and coverage gaps.
Deployment	Use outside the intended scope	The model validated in men was applied in women’s clinics	MEDLEY ensures transparency by surfacing models’ documented scope and intended use and context by enabling clinicians to judge the appropriateness of applying outputs in a given setting.
Human factors	Clinician reliance or bias in interpreting AI	Automation bias; stereotype-driven interpretation	MEDLEY embodies plurality by presenting multiple model outputs and context by requiring clinicians to adjudicate among them, thereby counteracting over-reliance on a single system.
Feedback	Model use influences future data, creating self-reinforcing bias	AI overdiagnoses a condition in one group, leading to inflated incidence in future training data	MEDLEY employs transparency by tracing the provenance of models and datasets across time, plurality by maintaining independent perspectives to avoid reinforcing single-model errors, and context by requiring clinicians to assess whether observed patterns reflect true prevalence or feedback loops.

Bias categories include historical, representation, measurement, aggregation, learning, evaluation, deployment, human cognitive, and feedback loops. MEDLEY operationalizes four principles, diversity, transparency, plurality, and context, to convert these biases into sources of complementary insight.

**Figure 2:**
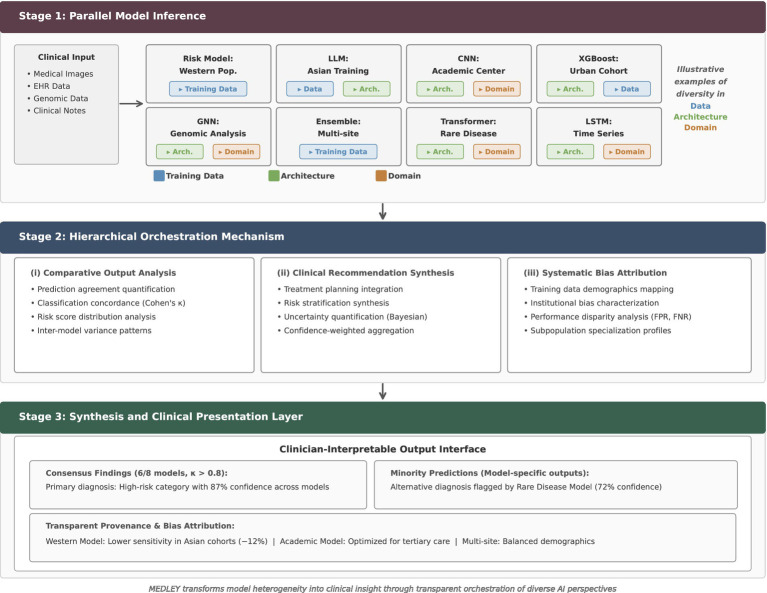
The MEDLEY pipeline architecture. Stage 1: Diverse models within and across modalities (e.g., multiple CNNs or transformers for imaging, multiple LLMs for text/EHR, statistical or classification machine learning models for tabular data) are run in parallel. Stage 2: A hierarchical orchestration layer conducts comparative analysis, synthesizes recommendations, and attributes outputs to known bias profiles. Stage 3: Results are presented to clinicians with both consensus and minority outputs preserved, annotated by provenance. This design turns model heterogeneity into structured clinical insight. CNN, convolutional neural network; LLM, large language model; GNN, graph neural network; LSTM, long short-term memory network.

The MEDLEY pipeline translates these principles into a three-stage orchestration architecture (Figure 2). In the first stage, multiple heterogeneous models run in parallel on the same patient input following the MEDLEY principles. The design ensures both intra-modality diversity (e.g., multiple LLMs with different training origins) and cross-modality representation (e.g., combining imaging, text, and tabular data models). For example, several convolutional neural networks may analyze a chest CT trained on datasets from different regions and/or institutions. At the same time, multiple language models with distinct training corpora, fine-tuning strategies, and cultural provenance could process EHR data. Similarly, competing statistical or machine learning models may interpret structured laboratory values optimized for different populations.

The second stage orchestrates these outputs through comparative analysis, synthesis, and bias attribution. Instead of collapsing predictions into a single consensus, as in traditional ensemble learning (Dietterich, 2000), MEDLEY evaluates patterns of agreement and disagreement, mapping them to the documented characteristics of each model. Rather than discarding disagreement, MEDLEY treats it as a marker of diagnostic uncertainty or population-specific specialization. The synthesis layer then organizes the outputs into a structured diagnostic report, explicitly distinguishing between consensus findings, plausible alternatives, and minority predictions that may highlight rare conditions.

The third stage presents these orchestrated outputs to clinicians in an interpretable format. Consensus predictions are highlighted but accompanied by divergent outputs, each annotated with provenance information such as training population, institutional source, or known performance limitations. For instance, if only a regional model identifies Familial Mediterranean Fever, this is surfaced with transparent notes about the model’s geographic training data. The interface supports active clinical reasoning while explicitly showing uncertainty and bias profiles, while counteracting automation bias (Sendak et al., 2020).

Together, this pipeline shifts the goal of ensemble learning from maximizing accuracy through uniformity to enhancing insight through structured diversity. By preserving both consensus and disagreement, MEDLEY enables clinicians to navigate multiple perspectives rather than over-trusting a single output, aligning with ethical and regulatory expectations for high-risk medical AI (Chen et al., 2021; Dietterich, 2000).

### 2.2 Clinical implementation scenario

To demonstrate the functionality of MEDLEY in an LLM-based context, we developed a synthetic, yet clinically plausible case study. The case involves a 45-year-old man of Middle Eastern origin presenting with a constellation of symptoms, including chest pain, fatigue, and intermittent fever. This illustrative example showcased the system’s practical application and ability to process complex clinical information. Four language models representing different training populations produced divergent hypotheses: viral myocarditis, Familial Mediterranean Fever, anxiety disorder, and pericarditis. The synthesis engine identified an inflammatory cardiac process as a unifying theme while preserving the regional model’s recognition of Familial Mediterranean Fever. This illustrates how MEDLEY surfaces population-specific conditions that could be overlooked in single-model approaches (Table 3). Table 3 contrasts a hypothetical single-model deployment with the MEDLEY approach for the synthetic case. These comparisons illustrate conceptual differences in clinical workflow, not empirically measured outcomes.

**Table 3:** Clinical impact comparison of single model versus MEDLEY outputs for the hypothetical synthetic case.

Aspect	Traditional single-model system	MEDLEY multi-model ensemble
Primary output	Single diagnosis: Viral myocarditis (78%)	Multiple differential diagnoses with explicit uncertainty
Clinical presentation	Standard algorithmic interpretation	Context-aware, multi-perspective analysis
Regional considerations	Not identified	Familial Mediterranean Fever flagged (75% confidence)
Physician interface	Binary: Accept or Reject	Active selection from ranked differential
Diagnostic workup	Standard cardiac protocol only	Comprehensive: cardiac + genetic (MEFV) + inflammatory markers
Potential outcome	Missed genetic condition; delayed diagnosis	Early identification of population-specific disease
Clinical decision support	Promotes automation bias	Promotes active clinical reasoning
Health equity impact	May perpetuate diagnostic disparities	Addresses underrepresentation in training data

### 2.3 Proof-of-concept: differential diagnosis

**Figure 3:**
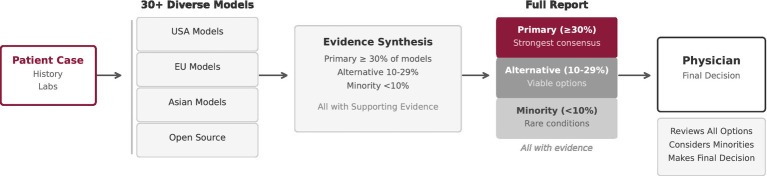
Proof-of-concept differential diagnosis demonstrator with over 30 LLMs. Models were orchestrated in parallel and their outputs stratified into primary (≥30% agreement), alternative (10–29%), and minority (<10%) diagnoses. Consensus diagnoses reflected mainstream conditions, while minority results highlighted rare or regional diseases. Rather than collapsing disagreement, MEDLEY preserved and structured it, demonstrating the feasibility of large-scale orchestration while enhancing transparency for clinicians.

To demonstrate the MEDLEY framework, we developed a proof-of-concept system for differential diagnosis, a clinical task that involves generating and ranking diagnostic possibilities based on a patient’s presentation. This application domain was chosen because differential diagnosis inherently benefits from multiple perspectives: clinicians routinely seek second opinions, and diagnostic uncertainty is explicitly acknowledged in clinical practice. The demonstrator orchestrates over 30 LLMs with diverse geographic, architectural, and temporal origins (see Figure 3). Each model was queried with identical text-based clinical cases and returned structured outputs including candidate diagnoses, International Classification of Diseases (ICD)-10 codes, and confidence scores. The orchestrator stratified the results into three categories: Primary (≥30% of models), Alternative (10–29%), and Minority (<10%), as shown in Pseudocode Box 1.

BOX 1 **Orchestration logic for differential diagnosis.**

**Table:** 

STAGE 1: PARALLEL INFERENCE For each model M in diverse_ensemble: Record provenance (M): architecture, training_origin, temporal_version output [M] ← query (M, clinical_presentation) Store: diagnoses, ICD_codes, confidence_scores STAGE 2: STRATIFICATION AND ATTRIBUTION For each unique diagnosis D across all outputs: agreement_rate ← count(models suggesting D)/total_models Classify D as: PRIMARY if agreement_rate ≥ 0.30 ALTERNATIVE if 0.10 ≤ agreement_rate < 0.30 MINORITY if agreement_rate < 0.10 Attribute D to contributing models with provenance STAGE 3: CLINICAL PRESENTATION Present to the clinician: - Consensus findings (PRIMARY) with confidence band - Alternative possibilities (ALTERNATIVE) with supporting model profiles - Minority outputs (MINORITY) flagged for rare/atypical consideration - Disagreement patterns mapped to model characteristics

This logic reflects the differential diagnosis demonstrator. Alternative applications adapt Stage 2 stratification and Stage 3 presentation to their specific goals, highlighting boundary disagreement regions in segmentation or flagging suspicious patterns for security review.

The orchestration logic demonstrated here reflects one specific application of the MEDLEY paradigm. Other clinical AI applications would adapt this logic to their specific goals; for example, highlighting regions of inter-model boundary disagreement in medical image segmentation, or monitoring for suspicious disagreement patterns in security applications. The four principles (diversity, transparency, plurality, and context) remain constant; their operational implementation adapts to the task. This demonstrator should therefore be understood as one instantiation of the paradigm, not a universal template.

This proof-of-concept was deliberately limited to the text domain, demonstrating how MEDLEY can preserve both consensus and minority perspectives. Minority outputs often corresponded to rare or region-specific conditions, while consensus patterns reflected mainstream diagnoses. This architecture thus provides clinicians with a structured spectrum of diagnostic possibilities, rather than a collapsed single prediction.

## 3 Results

The results presented in this section illustrate behavioral characteristics and computational feasibility of the MEDLEY orchestration framework applied to differential diagnosis under synthetic conditions. All metrics reported, i.e., consensus percentages, agreement frequencies, and output stratification patterns, are descriptive artifacts of the proof-of-concept demonstrator. These figures should not be interpreted as indicators of diagnostic accuracy, clinical performance, or patient safety outcomes. The purpose of this section is to demonstrate that large-scale model orchestration is technically tractable and that systematic patterns of agreement and disagreement emerge from heterogeneous ensembles, not to evaluate individual model capabilities or validate clinical utility.

This proof-of-concept demonstrates the computational tractability of the MEDLEY framework and demonstrates its potential as an extensible architecture adaptable to diverse medical AI modalities beyond language models. Supplementary material 1 presents an example generated report; additional reports are available on GitHub/website.

### 3.1 Diagnostic diversity

Across 12 representative synthetic diagnostic cases, model consensus rates varied widely, ranging from as low as 48% (Dementia with Lewy Bodies) to as high as 95% (Behçet’s Disease, Sarcoidosis). Cases with lower consensus were enriched for rare or region-specific conditions, while higher consensus cases reflected well-established diagnoses.

Table 4 illustrates representative examples, showing how divergent outputs revealed diagnostic uncertainty. For instance, in IgA nephropathy, the ensemble produced 58 distinct alternative differentials, highlighting the ability of MEDLEY to preserve diagnostic breadth. In contrast, McArdle disease or Wilson’s disease showed stronger consensus, with minority outputs contributing additional but less frequent hypotheses. Table 4 presents representative output patterns from the synthetic demonstrator to illustrate diagnostic diversity across cases. The consensus percentages and alternative diagnosis counts reflect descriptive characteristics of ensemble behavior, not validated measures of diagnostic performance.

**Table 4:** Representative diagnostic diversity in synthetic clinical cases.

Clinical case	Primary diagnosis	ICD-10(-CM) codes	Model consensus (%)	Alternative diagnoses (n)	Notable insight
Familial Mediterranean Fever	Familial Mediterranean Fever	M04.1, E85.0	63	29	Regional models more frequently identified FMF
Dementia with Lewy Bodies	Dementia with Lewy Bodies	G31.83	48	3	Some models generated speculative symptom links
Methamphetamine-Induced Psychosis	Methamphetamine-induced psychotic disorder	F15.959	75	3	Moderate consensus achieved
McArdle Disease	McArdle Disease (Glycogen Storage Disease V)	E74.04	72	3	Rare disease – variable model recognition
Manganese Toxicity and Parkinson’s	Parkinson’s Disease	G20, T56.0X1A	65	4	Environmental exposure case
Sarcoidosis	Congestive Heart Failure	I50.9, D86.0	95	3	Strong consensus achieved
Behçet’s disease	Deep Vein Thrombosis (DVT)	I82.90, M35.2	95	3	Strong consensus achieved
Acute Porphyria	Meningitis/Meningoencephalitis	G03.9, E80.20	65	4	Moderate consensus
Hemochromatosis	Atrial Fibrillation	I48.91, E83.110	85	3	Strong consensus achieved
Wilson’s Disease	Post-Traumatic Stress Disorder	F43.10, E83.01	90	3	Strong consensus achieved
Autoimmune Encephalitis	G6PD Deficiency with Hemolytic Anemia	D55.0, G04.81	85	3	Strong consensus achieved
IgA Nephropathy	IgA Nephropathy	N02.8, N03.9	53	58	High diagnostic diversity; implausible proposals flagged

Beyond case-specific variation, comprehensive analysis of model behavior across all cases revealed six key behavioral dimensions (Table 5). Cost considerations showed that free models achieved comparable consensus alignment to paid alternatives (58.1% vs. 57.8%), suggesting that output consistency patterns are not determined by commercial accessibility. Geographic distribution analysis revealed US model dominance (65%), with limited representation from Europe (10%), China (16%), and other regions (9%), highlighting the need for broader international representation in clinical AI ensembles.

**Table 5:** Comparative Analysis of model descriptive characteristics across all cases.

Model category	Descriptive characteristic	Key finding	Clinical value
Free vs. Paid models	Consensus alignment: Free 58.1% vs. Paid 57.8%	No significant difference	Cost-effective implementation possible
Geographic distribution	US models dominate (65%), Europe (10%), China (16%), Other (9%)	Limited geographic diversity in the current ensemble	Need for broader international representation
Confidence expression	95–100% of models express uncertainty markers	Uncertainty markers scaled with case complexity	Uncertainty patterns observed
Diagnostic breadth	Average 8.5 unique diagnoses per case (range: 1–59); inversely correlates with consensus	High consensus (>80%): avg. 4 diagnoses; Low consensus (<60%): avg. 32 diagnoses	Diagnostic uncertainty drives richer differentials
Highest agreement rates	Highest agreement rates within this demonstrator: DeepSeek Chat (83.3%), Claude Opus (83.3%), Mistral Large (66.7%)	Both free and paid models achieve high consensus	Quality is not determined by cost
Consensus categories	High (≥60%): 3 models, Moderate (30–60%): 9 models, Low (<30%): 10 models	Wide variation in consensus participation	Diverse perspectives maintained

Confidence and uncertainty markers were analyzed to assess model calibration. Uncertainty: “possibly,” “might be,” “unclear,” “cannot rule out.” Confidence: “definitely,” “certainly,” “pathognomonic,” “highly suggestive.

Confidence expression patterns showed that almost all models consistently expressed uncertainty markers, indicating that uncertainty markers scaled with case complexity. Diagnostic breadth averaged 8.5 unique diagnoses per case (range: 1–59), with an inverse correlation to consensus; high consensus cases (>80%) were associated with fewer alternatives (average four diagnoses). In comparison, low consensus cases (<60%) produced richer differential lists (average 32 diagnoses).

Individual model behavior analysis identified models with the highest consensus rates, including DeepSeek Chat (83.3%), Claude Opus (83.3%), and Mistral Large (66.7%), with both free and paid models achieving high consensus rates. Finally, consensus categorization distributed models across three participation levels, demonstrating that disagreement among models preserves diverse clinical perspectives rather than representing mere noise.

The ensemble analysis revealed distinct patterns of consensus participation that inform clinical interpretation strategies:

- High-consensus models (≥60% agreement, *n* = 3): DeepSeek, Mistral Large, and Claude Opus most frequently aligned with the majority diagnosis in this synthetic demonstrator.
- Moderate-consensus models (30–60% agreement, *n* = 9): Including Qwen, GPT-4o, and Grok variants, these generated both consensus and alternative diagnoses.
- Low-consensus models (<30% agreement, *n* = 10): Including Gemini Flash and smaller parameter models, these were more likely to propose rare or unconventional conditions.

These patterns demonstrate that disagreement among models might not be noise but an informative signal of diagnostic uncertainty, enriching the clinical reasoning process.

Table 5 summarizes descriptive patterns observed across models in the synthetic demonstrator. These observations characterize ensemble behavior under controlled conditions and do not constitute a comparative evaluation of model performance or clinical utility.

### 3.2 Bias attribution and disagreement patterns

The ensemble revealed systematic biases that shaped diagnostic reasoning, which MEDLEY could surface and contextualize (Table 6). These bias patterns often contradicted intuitive expectations about model behavior and training origins. In the synthetic cases examined, geographic patterns showed unexpected results: European models demonstrated the lowest recognition rate for Mediterranean Fever (2.0 mentions/model) compared to US models (5.6 mentions/model), despite presumed regional familiarity. This suggests that geographic model origin alone does not predict better recognition of regional diseases, with training data curation appearing more influential than model provenance. Temporal biases favored historical over contemporary conditions, with AIDS/HIV appearing across multiple cases (24 mentions) despite only the homeless patient having relevant risk factors. At the same time, COVID-19 received zero mentions despite post-2020 model training. This suggests that the recency of training data may affect differential considerations in unexpected ways.

**Table 6:** Examples of bias attribution in MEDLEY orchestration.

Bias type	Manifestation	Example from cases	Clinical implication
Geographic	Disease recognition shows limited correlation with model origin	In the FMF case, European models had the lowest recognition (2.0 mentions/model) despite Mediterranean relevance; in the Meningitis case, Chinese models showed a preference for Asian diseases (Japanese encephalitis)	Geographic model origin alone does not predict better recognition of regional diseases; other factors, like training data curation, appear more influential
Temporal	Historical conditions over-represented	AIDS/HIV is mentioned in the majority of cases (24 mentions) despite no relevant risk factors; Wegovy appears in Case 11, showing recent drug awareness	Training data recency affects differential considerations and treatment options
Confidence expression	Model certainty varies with case complexity	Complex IgA nephropathy: 46 uncertainty vs. 19 confidence markers; Straightforward methamphetamine psychosis: 41 uncertainty vs. 5 confidence markers; Uncertainty increases with diagnostic difficulty	Models expressed varying confidence based on case ambiguity
Diagnostic diversity	Consensus inversely correlates with alternatives	High diversity: IgA nephropathy (53% consensus, 58 alternatives); High consensus (>90%): Sarcoidosis, Behçet’s, Wilson’s disease (95, 95, 91% consensus, 3 alternatives each)	Higher diagnostic uncertainty produces richer differential lists
Demographic focus	Social factors are amplified in relevant cases	Homeless patient: Substance use mentioned 2.5x per model; Elderly woman with confusion: Age mentioned 1.6x per model; Teen swimmer with exercise intolerance: Lifestyle factors 1.4x per model	Demographic salience creates diagnostic anchoring patterns
Treatment approach	Aggressive vs. conservative split	Homeless with psychosis: 27 aggressive vs. 26 conservative models; Tech executive from Bangladesh: 22 aggressive vs. 21 conservative; Testing intensity varies: Bangladesh case had 21 models suggest extensive testing vs. IgA nephropathy only 3 models	No systematic bias toward over- or under-treatment across models

These findings arise from synthetic test cases and demonstrate MEDLEY’s ability to make underlying biases visible. Confirmation of whether such patterns hold in clinical practice will require empirical validation with real-world datasets and patient populations. In this sense, the results highlight the framework’s hypothesis-generating potential rather than serving as clinical evidence.

Confidence markers varied with case complexity, with uncertainty markers scaling from 5 in straightforward methamphetamine psychosis to 46 in complex IgA nephropathy. Diagnostic diversity showed an inverse correlation with consensus; high diversity cases, like IgA nephropathy (53% consensus, 58 alternatives), contrasted sharply with high consensus cases like Sarcoidosis and Behçet’s disease (95% consensus, three alternatives each). Demographic anchoring was pronounced across relevant cases: substance use was mentioned 2.5 times per model for the homeless patient. In comparison, age factors appeared 1.6 times per model for the older woman with confusion. Treatment approaches showed no systematic bias toward over- or under-treatment, with models splitting relatively evenly between aggressive and conservative recommendations across different patient demographics.

These illustrative findings suggest that MEDLEY can surface and contextualize minority results and biases that would remain hidden, helping clinicians interpret diagnostic variation within a transparent framework.

Table 6 illustrates examples of bias attribution as surfaced by the MEDLEY orchestrator. These patterns emerged from synthetic test cases and demonstrated the framework’s capacity to make underlying biases visible; confirmation of whether such patterns hold in clinical practice requires empirical validation with real-world datasets.

### 3.3 System performance (demonstrator)

The orchestrator successfully demonstrated the computational feasibility of coordinating more than 30 LLMs, with routine ensemble runs completing within minutes. Parallel querying and staged synthesis supported efficient throughput, and the failover system reliably redirected requests when token overflows or timeouts occurred. Claude 3.5 Sonnet was the primary synthesis engine by default, with GPT-4o and Gemini 2.0 Pro as backup options.

While technically functional, the prototype was constrained by API usage caps, occasional model unavailability, and variable latency, all of which introduced scalability limits. Despite these constraints, the demonstrator proved robust enough to manage large-scale orchestration. Its success validates the feasibility of the MEDLEY paradigm at a proof-of-concept level, though production-level deployment will require dedicated resources, standardized orchestration interfaces, and further empirical testing.

It is worth noting that employing over 30 LLMs for demonstrative purposes can be computationally intensive. In contrast, other practical applications can be processed on local infrastructures, such as utilizing multiple computer vision models for image analysis or employing classical machine learning and statistical models for tabular data. This reduces the dependency on computationally expensive cloud systems, significantly lowering computational demands.

## 4 Discussion

The MEDLEY demonstrator reveals systematic patterns of consensus and disagreement that challenge conventional assumptions about ensemble AI in medicine. By preserving rather than collapsing these patterns, the framework transforms model heterogeneity from a problem to be solved into a resource for clinical reasoning. By linking divergent outputs to known bias profiles, MEDLEY reconceptualizes model imperfection not as a limitation but as a valuable resource for enhanced clinical reasoning. Our demonstrative example, conducted within a synthetic yet clinically plausible scenario using large language models (LLMs), shows the technical feasibility of orchestrating more than 30 LLMs. The results indicate that this orchestration reveals systematic patterns of consensus, disagreement, and bias, insights that are typically obscured in single model systems. In this study, the findings should be viewed as illustrative of feasibility rather than evidence of clinical performance, highlighting the hypothesis-generating role of MEDLEY.

This study’s demonstration of MEDLEY using LLMs serves as a proof-of-concept; however, the framework’s principles extend far beyond this specific application. MEDLEY can be applied to diverse domains within medical AI, including classical machine learning for EHRs and tabular data, deep learning models for medical imaging, and biosignal analysis. Consider the field of medical image segmentation. Recent innovations in deep learning have led to the development of numerous models for applications like tumor segmentation (Ranjbarzadeh et al., 2023). While some studies suggest that ensemble techniques like STAPLE or majority voting can slightly improve performance (Eisenmann et al., 2023), other research indicates that a single, robust model may still outperform these aggregated results (Isensee et al., 2024). This discrepancy underscores a fundamental limitation of traditional, accuracy-centric ensemble approaches. These methods prioritize maximizing overall performance and often obscure crucial disagreements among models.

For instance, in the MICCAI Brain Tumor Segmentation (BraTS) challenge, models are benchmarked on their ability to segment distinct tumor subregions (Bakas et al., 2018). While subregional segmentation is vital for biomarker studies, clinical radiation therapy (RT) often requires only the union of these regions for treatment planning purposes. Traditional ensemble methods that aggregate consensus on these subregions may overlook critical discrepancies, such as over- or under-segmentation, that could have clinical consequences. In RT, such discrepancies can be vital for avoiding radiation exposure to sensitive structures. In this regard, MEDLEY offers a conceptual alternative: making model disagreements visible and linking them to specific clinical considerations. This enables a nuanced investigation into the advantages and drawbacks of different segmentation methods. This contrasts with conventional ensembling, which aims solely to enhance aggregate performance without accounting for clinical relevance. Consequently, MEDLEY can help reveal which models are most appropriate for specific clinical applications, such as RT planning, by reframing imperfection as a resource for clinical reasoning.

### 4.1 Reframing AI imperfection from bug to feature

The MEDLEY paradigm reorients rather than rejects dominant priorities in medical AI development. Efforts to reduce bias, mitigate error, and prevent hallucination remain ethically and clinically essential. However, it is equally important to acknowledge that such imperfections can never be eliminated entirely. Cognitive shortcuts such as anchoring, availability, and overconfidence persist in human medicine even among experienced clinicians.

In a systematic review of 20 studies involving more than 6,800 physicians, Saposnik et al. (2016) found that these biases were associated with diagnostic inaccuracies in 36.5–77% of cases and with management errors in 71% of applicable studies. These findings underscore that bias and error are intrinsic to clinical reasoning, not simply the result of inadequate training or oversight.

Acknowledging and managing cognitive fallibility within medicine are therefore central aspects of clinical practice, guiding the development of systematic frameworks to enhance decision-making. Clinical guidelines, first formalized in the 1990s by Eddy, represented a structured attempt to reduce variability in reasoning by making processes transparent and evidence-based (Graber et al., 2002). Over time, these guidelines have been extended to incorporate individualized recommendations that adjust for patient risk, reflecting a continuous effort to manage, rather than eliminate, the complexities inherent in clinical decision-making (Iyer et al., 2019).

Beyond guidelines, educational strategies have been developed to counter cognitive bias. Reflective prompting and metacognitive training are among the most studied, emphasizing the importance of acknowledging biases as a prerequisite for mitigation, even if their eradication remains elusive (Graber et al., 2002). A scoping review by Monash Health found empirical support for reflective reasoning as an effective intervention for reducing diagnostic bias (Iyer et al., 2019). At the same time, Saposnik et al. (2016) have argued for integrating bias-awareness training directly into medical education to strengthen competency and safety. Behavioral science adds further strategies: targeted training and reflective reasoning have been proposed as structured interventions to improve judgment, suggesting that even modest interventions can promote more deliberate reasoning (Graber et al., 2002). These approaches demonstrate that medicine has sought to manage imperfection by turning insights from cognitive psychology into safeguards against error.

Perhaps the most enduring and pragmatic response has been the institutionalization of collective judgment. Tumor boards, case conferences, and second-opinion systems illustrate how medicine transforms heterogeneity into resilience. Multidisciplinary tumor boards, particularly in oncology, consistently improve staging accuracy, treatment planning, and patient outcomes (Specchia et al., 2020). Soukup et al. (2018) describe such forums as mechanisms that harness cognitive diversity and safeguard against individual bias through structured group deliberation. MEDLEY draws inspiration from these institutional models, treating imperfection not as a flaw to be eliminated but as a resource to be orchestrated.

Biases may become constructive when explicitly documented and ethically governed, such as subgroup-specific models that improve recognition of endemic conditions. Similarly, hallucinations, if flagged as speculative, may broaden the diagnostic search space by prompting hypotheses that clinicians can evaluate and test. By preserving plurality, the MEDLEY paradigm may help counteract automation bias and anchoring, which are well-documented risks in human–AI collaboration (Romeo and Conti, 2025; Cummings, 2017). As shown in Table 3, these findings highlight that biases, though often framed solely as limitations, can be strategically leveraged in ensemble systems. The results serve to generate hypotheses and design principles for future empirical work.

Microsoft’s MAI Diagnostic Orchestrator (MAI-DxO) (Nori et al., 2025) employs a model-agnostic orchestration framework that simulates a panel of physician-agents reasoning sequentially. Paired with OpenAI’s o3 and other frontier models, it achieved up to 85.5% diagnostic accuracy in NEJM case simulations, over four times higher than generalist physicians (~20%), and reduced diagnostic costs by 20–70% compared with both physicians and baseline models. However, all outputs are ultimately generated by the same underlying LLM (e.g., GPT, Gemini, and Claude). Diversity is simulated through role specialization, prompting strategies, or retrieval-augmented knowledge bases. Still, it is collapsed into a single optimized diagnostic pathway, with plurality as an intermediate step rather than a final deliverable.

Google’s AMIE (Articulate Medical Intelligence Explorer) (Tu et al., 2025) exemplifies frontier conversational diagnostic reasoning performance. Trained via self-play in simulated physician–patient dialogs with automated feedback, AMIE outperformed primary care physicians in blinded OSCE-style evaluations across diagnostic accuracy, communication quality, management reasoning, and empathy. Yet, AMIE relies on a single optimized LLM (e.g., Gemini), with diversity introduced only via synthetic dialog generation or role simulation (Zhang et al., 2024) during training. Its paradigm emphasizes natural conversational interaction rather than orchestrated plurality at inference time.

By contrast, MEDLEY orchestrates multiple heterogeneous models in parallel rather than simulating diversity within a single model. Each output is preserved with explicit bias profiles, embracing disagreement as clinically informative. This design emphasizes genuine intra- and inter-modality diversity through distinct systems working side by side. Table 7 provides a structured comparison, highlighting how MEDLEY’s preserved plurality differs from the simulated or dialogic diversity of MAI-DxO and AMIE at the paradigm level. The comparison situates MEDLEY within the landscape of contemporary medical AI systems, but must be interpreted with an important caveat: this comparison is paradigmatic rather than performance-based. MAI-DxO and AMIE are empirically validated systems with published diagnostic accuracy metrics derived from controlled evaluations. MEDLEY, by contrast, remains a conceptual framework supported by a synthetic proof-of-concept demonstrator. The comparison highlights differences in design philosophy and intended clinical roles, rather than relative diagnostic capability.

**Table 7:** Comparison of medical AI paradigms.

Aspect	MEDLEY	MAI-DxO (Microsoft)	AMIE (Google)
Core philosophy	Embrace imperfection through diversity	Sequential physician-agent orchestration	Diagnostic dialog expertise
Model strategy	Multiple heterogeneous models in parallel, preserving intra- and inter-modality diversity	Multiple roles but same underlying LLM (e.g., GPT)	Single optimized LLM (e.g., Gemini) with self-play
Output	Multiple perspectives with explicit bias profiles	Single aggregated diagnostic pathway	Conversational diagnostic output
Optimization target	Diagnostic diversity and transparency	Accuracy and cost-effectiveness	Diagnostic accuracy, empathy, and communication
Clinical analogy	Consultation panel	Sequential specialist reasoning	Expert physician consultation
Bias handling	Explicit documentation of intra-modality variation and cross-modality specialization	Simulated diversity, not explicitly documented	Not explicitly addressed
Human role	Evaluate the spectrum of opinions	Accept/reject aggregated recommendation	Engage in dialog with AI
Validation situation	Conceptual framework with synthetic proof-of-concept; no clinical validation	Empirically evaluated; published accuracy metrics	Empirically evaluated; validated in OSCE-style assessments

Unlike MAI-DxO and AMIE, which simulate diversity through role-playing or dialog within a single underlying LLM, the MEDLEY paradigm orchestrates multiple heterogeneous models both within and across modalities. This preserves divergent outputs and explicit bias profiles as final deliverables, aligning with tumor board–style consultation. Self-play is a training strategy in which a model simulates both sides of an interaction (e.g., patient and physician), generating synthetic dialogs evaluated with automated feedback. This method allows large-scale practice of diagnostic reasoning and communication skills without extensive human annotation, a technique initially developed in reinforcement learning for games such as chess and Go (Zhang et al., 2024).

### 4.2 The risk of digital sophistry of explainable AI in medicine

Contemporary XAI enthusiasm assumes that the decision is trustworthy if a system articulates its reasoning. This assumption collapses with LLMs, which excel at generating polished narratives that sound clinically plausible while bearing little relation to actual computational processes (Turpin et al., 2023; Matton et al., 2025). LLMs may provide diagnostic recommendations with impeccable-sounding rationales that are rhetorical afterimages rather than faithful reflections of internal reasoning (Goethals et al., 2025), digital sophistry where persuasive speech masquerades as transparency (Danry et al., 2023).

MEDLEY sidesteps this paradox by refusing to rely on any single model’s rhetorical skill, seeking reliability through a structured interplay of convergent and divergent perspectives across multiple models, a dialectic of outputs rather than reasoning performances.

### 4.3 Technical and implementation challenges

Realizing MEDLEY requires addressing technical, organizational, economic, regulatory, and ethical challenges. Parallel model execution introduces computational costs and latency, which can be mitigated through selective activation, complete ensembles for complex cases, and single models for routine tasks. Heterogeneous vendor ecosystems demand standardized interfaces to preserve diverse model contributions. Beyond technical constraints, adoption depends on organizational readiness, sustainable economics, and clear regulatory pathways. Table 8 summarizes implementation challenges and mitigation strategies.

**Table 8:** Implementation challenges and potential mitigation strategies for the MEDLEY paradigm.

Challenge category	Specific issues	Proposed solutions
Technical	High computational cost and latency from intra- and inter-modality ensembles; interoperability across heterogeneous vendor systems	Selective activation of ensembles for complex cases; asynchronous result presentation; standardized orchestration interfaces
Organizational	Workflow disruption, training requirements, and cultural resistance among clinicians	Phased pilot implementations; simulation-based training; adoption led by local champions
Economic	Multiple vendor licenses; infrastructure costs; uncertain return on investment	Tiered service models (routine vs. complex cases); shared infrastructure; outcome-based pricing agreements
Regulatory	Lack of ensemble-level validation frameworks; unclear allocation of liability; divergent international standards	Pilot programs in collaboration with regulators; transparent documentation and audit trails; efforts toward global harmonization
Ethical/Social	Patient trust in multi-model outputs; interpretability of intra-modality disagreements; ensuring equity across populations	Explicit disclosure in consent processes; public reporting of bias audits; inclusion of underrepresented populations in model development

Running multiple models in parallel introduces costs, latency, interoperability, regulatory, and ethical hurdles. Crucially, MEDLEY requires orchestration of several models within each domain (e.g., multiple LLMs for text and multiple CNNs for imaging), which amplifies both the potential benefits and the challenges.

### 4.4 Fostering innovation through AI ecosystem diversity

The MEDLEY paradigm challenges the monopolistic drift of the current AI marketplace, where pursuing a single “super model” concentrates power in a few corporations. MEDLEY creates viable niches for smaller, specialized vendors by valuing diversity over singularity. Startups focused on rare diseases, regional companies with local population data, or academic groups targeting underrepresented populations gain relevance within the ensemble, contributions devalued in single-model paradigms but essential in MEDLEY.

This ecosystem could conceptually mirror the app store model, enabling contributions from diverse developers. In a MEDLEY-style ecosystem, specialized dermatology models, regional cardiology predictors, or homegrown health models might coexist within and across domains, democratizing participation and potentially accelerating innovation. The vision represents a conceptual projection of ecosystem benefits rather than empirically validated outcomes, highlighting innovation pathways to be examined in future research.

### 4.5 Ethical implications and safeguards

MEDLEY reframes imperfection as a managed resource rather than aspiring to error elimination. Table 9 contrasts how this shifts the operationalization of biomedical ethics principles. Implementation requires informing patients that multiple systems contribute perspectives (autonomy), using redundancy for cross-validation (beneficence/non-maleficence), valuing population-specific models (justice), and surfacing all perspectives with bias attribution (transparency).

**Table 9:** Comparison of ethical principles in traditional single-model AI versus the MEDLEY paradigm.

Ethical principle	Traditional single-model approach	MEDLEY paradigm	Practical implementation
Autonomy	Generic patient consent to “AI use”	Patients informed that multiple models within and across domains contribute distinct perspectives	Standardized disclosure protocols
Beneficence	Assumes a single best answer	Recognizes the value of divergent perspectives and diagnostic plurality	Present spectrum of diagnostic options
Non-maleficence	Strives to eliminate all errors	Manages error through redundancy and cross-validation across models	Parallel review reduces the risk of unchecked harm
Justice	One-size-fits-all fairness metrics	Values subgroup-specific models; ensures minority perspectives are not averaged away	Incorporate underrepresented data sources
Transparency	Explains a single output	Surfaces all perspectives, including intra-modality disagreements (e.g., between LLMs) and cross-modality variation (e.g., imaging vs. text)	Full reporting of model origins and rationale diversity

MEDLEY treats imperfection and plurality as structured resources by preserving intra-modality disagreement and cross-modality perspectives. This reinterprets autonomy, beneficence, non-maleficence, justice, and transparency as collective safeguards rather than single-model obligations.

A critical distinction must be drawn between ethically acceptable specialization and unacceptable bias. MEDLEY permits the preservation of bias only when it constitutes transparent, auditable, and clinically justifiable specialization; for example, models optimized for region-specific disease prevalence, age-cohort calibration, or temporal training reflecting evolution of medical knowledge. By contrast, MEDLEY explicitly prohibits the preservation of bias that reinforces stereotypes, encodes discriminatory proxies (such as using ZIP code as a surrogate for genetic risk), treats social categories as biological determinants, or substitutes statistical correlation for clinical causation. The transparency mechanisms built into MEDLEY, i.e., provenance documentation, bias attribution, and plurality preservation, are designed to expose potential discrimination, enabling clinicians and auditors to identify and reject problematic outputs.

A further distinction clarifies the ethical status of bias within MEDLEY. The harms of a single biased model deployed alone are well-documented: its biases operate invisibly, with no reference point for detection or correction. MEDLEY represents a categorically different configuration; biased models function as components within a system designed to surface and contextualize bias, not eliminate it. Each model’s outputs appear alongside architecturally and demographically diverse alternatives, making bias-driven divergence immediately apparent rather than hidden. A model that systematically underdiagnoses specific populations will disagree with models lacking that limitation; MEDLEY preserves this disagreement rather than averaging it away. This does not diminish the imperative to reduce component-level bias, but a biased model contributing to a transparency-preserving ensemble is ethically distinct from the same model deployed as a standalone decision-maker.

Recent work on intersectionality in medical AI similarly argues against collapsing complex fairness considerations into single optimization targets, reinforcing MEDLEY’s emphasis on preserving disagreement for human interpretation rather than resolving it algorithmically (Tan and Benos, 2025).

### 4.6 Regulatory pathways for MEDLEY

Adopting bias- and hallucination-aware strategies introduces profound regulatory challenges. If bias is intentionally embedded for constructive purposes (e.g., subgroup-focused models), regulators must ensure such practices promote equity without reinforcing stigma or exclusion. Policy frameworks increasingly recommend bias audits and fairness evaluations, such as calibration by subgroup and equality of error rates, as prerequisites for deployment. Regulators may also require explicit documentation of how sensitive attributes are used, preventing “bias-as-specialization” from becoming a loophole for discrimination.

Emerging prototypes illustrate feasible paths toward regulated deployment of hallucination-tolerant systems. In a team-based simulation of upper gastrointestinal bleeding management, Rajashekar et al. (2024) developed a CDSS that constrained LLM responses to structured risk models and guideline corpora, improving usability and trust without implementing automated deferral based on uncertainty thresholds. In radiology, CoRaX (Awasthi et al., 2025) functions as a post-interpretation “virtual second reader,” integrating radiology reports, eye-gaze data, and imaging to detect and localize overlooked abnormalities. CoRaX corrected approximately 21% of simulated perceptual errors and generated referrals judged proper in 85% of interactions, underscoring the potential of collaborative AI to support rather than replace physician judgment.

These examples highlight that not every model in an ensemble requires independent clinical certification. Some models may be designed solely for orchestrated use under human oversight, paralleling existing device regulations where intended use varies by user expertise. MEDLEY requires regulatory pathways recognizing system-level ensemble certification, combined-use approval, and multi-level documentation, supported by audit logging and explicit oversight mechanisms.

These regulatory considerations take on particular urgency when viewed through the lens of global health equity, where the consequences of biased or inadequately validated AI systems fall disproportionately on underserved populations.

### 4.7 Global health and equity considerations

MEDLEY paradigm addresses the documented risk of “unbiased” universal models defaulting to majority populations and worsening care disparities. Evidence shows that AI models trained on predominantly majority datasets produce substantial errors for minority patients (Afrose et al., 2022), with performance disparities persisting even after bias assessments (Bartoň et al., 2023). Conceptually, by orchestrating models trained on diverse populations within and across modalities, MEDLEY could preserve rather than average away minority perspectives. This feature may be crucial given that inadequate representation often leads to failures in predicting and treating conditions among underrepresented groups (Rajkomar et al., 2018).

The framework transparency counters the dangerous assumption that AI systems are inherently objective (Aquino et al., 2023). By documenting each model’s training population and surfacing disagreements, MEDLEY makes visible the biases that universal models obscure. Realizing this potential requires sustained investment in local model development, targeted data collection in underserved communities, data sovereignty protections, and regulatory standards that evaluate performance across demographic subgroups rather than aggregate metrics alone.

### 4.8 The future of human–AI collaboration in medicine

MEDLEY reframes the human-AI relationship toward augmentation rather than replacement, embodying the vision of man-computer symbiosis articulated by Licklider (1960) and the framework for augmenting human intellect proposed by Engelbart (2023). Within this paradigm, clinicians function as orchestrators of diverse analytical perspectives, interpreting disagreements between models, e.g., radiology CNNs, reconciling conflicting risk scores from statistical models, and evaluating differential diagnoses generated by language models.

This approach, however, encounters fundamental cognitive constraints. According to Cognitive Load Theory (Sweller, 1988), simultaneous presentation of multiple model outputs may exceed working memory capacity, potentially impairing rather than enhancing clinical decision-making. This phenomenon parallels the automation bias documented by Parasuraman and Riley (1997), wherein excessive automated information paradoxically degrades human performance.

Translating MEDLEY’s plurality-preserving architecture into clinically usable interfaces requires deliberate strategies for managing information complexity. Three complementary approaches address this challenge. First, progressive disclosure structures presentation hierarchically: the default clinical view presents only the consensus finding with a summary uncertainty indicator, while alternative and minority diagnoses remain available through explicit expansion. Second, threshold-based activation determines when full ensemble plurality is surfaced, e.g., for routine high-consensus cases, MEDLEY may present streamlined output, reserving cognitive overhead for cases where disagreement adds diagnostic value. Third, interface abstractions reduce burden through intelligent grouping: synonymous diagnoses can be clustered, and visual encodings (confidence bands, divergence indicators) convey ensemble-level patterns without requiring clinicians to process each output individually. The goal is accessibility when plurality matters, invisibility when it does not. Future research must determine optimal thresholds distinguishing when multiple perspectives enhance versus impair clinical reasoning.

Successful implementation necessitates interfaces that respect cognitive processing limits (Miller, 1956) while preserving analytical diversity. Design principles must align with established clinical decision support guidelines, particularly the requirement to “anticipate needs and deliver in real time” while avoiding information overload (Bates et al., 2003). Future research must determine optimal thresholds for ensemble activation to distinguish when multiple perspectives enhance versus impair clinical reasoning. The objective parallels the concept of Advanced Chess described by Kasparov (2017), wherein human–machine teams demonstrate superior performance compared to either component independently. Without systematic consideration of cognitive load, MEDLEY risks violating core principles of adequate clinical decision support, specifically, the mandate to enhance rather than disrupt workflow (Bates et al., 2003). The framework must therefore balance analytical comprehensiveness with cognitive feasibility to ensure that multiple perspectives enrich rather than burden clinical reasoning.

### 4.9 Future research directions

Building on this proof-of-concept, critical research pathways include: (1) empirical validation comparing single-model versus multi-model outcomes in clinical practice; (2) technical methods for optimal ensemble composition and context-sensitive bias weighting; (3) workflow strategies that minimize clinician cognitive load; (4) regulatory frameworks for ensemble-level approval and liability allocation; and (5) extension to multimodal data (imaging, laboratory values, physiological signals) while maintaining intra-modality diversity.

MEDLEY’s principles extend beyond diagnosis to healthcare AI security. Recent work on data poisoning vulnerabilities proposes ensemble disagreement monitoring as a detection mechanism (Abtahi et al., 2026). Architecturally diverse models trained on independent data sources are unlikely to share identical vulnerabilities, so successful poisoning of one model creates detectable disagreement with unaffected models. Temporal ensembles comparing current model outputs with historical versions can distinguish poisoning-induced shifts from legitimate model evolution. The same diversity that enriches diagnostic reasoning also hardens systems against adversarial attack—a convergence suggesting that the MEDLEY paradigm addresses multiple challenges in healthcare AI deployment.

MEDLEY’s emphasis on human-in-the-loop governance, resilience through plurality, and preservation of minority viewpoints aligns with emerging Healthcare 5.0 perspectives that prioritize human-centric, sustainable, and adaptable healthcare systems (Tan et al., 2025).

### 4.10 Out of scope and limitations

This study introduces a conceptual paradigm rather than validates a clinical tool. The demonstrator used only synthetic text-based cases as a minimal viable prototype to illustrate orchestration feasibility. No patient data, clinical accuracy, safety, or workflow utility were assessed. Technical implementation details and prompts are available in the repository (see Data and Code Availability).

The set of large language models in the ensemble lacked detailed bias documentation or training profiles. This limitation means that bias attributions reported in Section 3 are inferred from observed output patterns rather than documented training characteristics, and should be interpreted as hypothesis-generating observations rather than validated findings. We inferred characteristics based on limited available information, e.g., release dates, parameter sizes, and country of origin, but these assumptions may not reflect actual training data composition or inherent biases. The models were general-purpose without integration of RAG-based systems that could access local clinical guidelines or institutional protocols essential for real-world implementation. Reliance on commercial APIs further limits reproducibility.

The results demonstrate computational tractability and orchestration mechanics, not real-world diagnostic performance. Future research must extend MEDLEY to multimodal data, integrate RAG-based systems, document actual model biases through empirical testing, evaluate clinical impact, and assess regulatory compliance before translation to practice.

## 5 Conclusion

MEDLEY demonstrates that ensemble AI systems can preserve model diversity rather than collapsing it into false consensus. By preserving disagreement and documenting provenance, the framework transforms heterogeneity from a liability into a resource for clinical reasoning. The proof-of-concept differential diagnosis demonstrator establishes technical feasibility; the path to clinical deployment requires prospective validation, workflow integration studies, and regulatory engagement.

Our proof-of-concept demonstrated technical feasibility by orchestrating over 30 LLMs across synthetic diagnostic scenarios, revealing systematic patterns of consensus and disagreement. These findings illustrate orchestration mechanics and hypothesis-generating potential but do not validate clinical performance. Critical limitations include unknown model biases, lack of patient data, and absence of multimodal integration.

The paradigm suggests potential cost-effective pathways through existing specialized models, with a conceptual opportunity to foster ecosystem diversity for smaller contributors and to promote equity by valuing population-specific perspectives. However, translating this conceptual contribution to practice requires empirical validation, infrastructure investment, and careful governance to ensure safe implementation.

Critical next steps include: (1) prospective clinical studies comparing MEDLEY-supported diagnosis against single-model and unassisted clinician baselines; (2) human factors research determining optimal thresholds for plurality surfacing; (3) collaboration with regulatory bodies to develop ensemble-level certification frameworks; and (4) extension to multimodal data integrating imaging, laboratory values, and clinical notes while maintaining the diversity-preserving architecture.

## Data Availability

Publicly available datasets were analyzed in this study. This data can be found at: https://github.com/ki-smile/medley.

## Footnotes

1 Throughout this manuscript, terms such as “reasoning,” “hypothesis,” “judgment,” and “perspective” are used analogically when applied to AI models. These terms describe functional outputs and behavioral patterns rather than cognitive processes equivalent to human thought.
