# Supplementary material for: Leveraging imperfection with MEDLEY: a multi-model approach harnessing bias in medical AI
Source: Front Artif Intell. 2026 Mar 4;9:1701665. doi: 10.3389/frai.2026.1701665 (PMC12997180; doi:10.3389/frai.2026.1701665)
Supplement: Supplementary file 1 [file Data_Sheet_1.pdf]

Supplementary Material

Leveraging Imperfection with MEDLEY A Multi-Model Approach Harnessing Bias in Medical AI

Farhad Abtahi[1,2,3*], Mehdi Astaraki[1], Fernando Seoane[1,3,4,5]

[1]Department of Clinical Science, Intervention and Technology, Karolinska Institutet, 17177 Stockholm, Sweden

[2] Department of Biomedical Engineering and Health System, School of Engineering Sciences in Chemistry, Biotechnology and Health, KTH Royal Institute of Technology, 14157 Huddinge, Sweden

[3] Department of Clinical Physiology, Karolinska University Hospital, 17176 Stockholm, Sweden

[4] Department of Textile Technology, Faculty of Textiles, Engineering and Business Swedish School of Textiles, University of Borås, 503 32 Borås, Sweden

[5] Department of Medical Technologies, Karolinska University Hospital, 141 57 Huddinge, Sweden

*** Correspondence:**
Farhad Abtahi
farhad.abtahi@ki.se

The clinical cases presented in this supplementary material are entirely fictitious.
All patient names, characteristics, and diagnostic details are generated for illustrative purposes and do not correspond to any real individuals.

MEDLEY

Medical AI Ensemble Clinical Decision Report

Case ID: Case_5	Title: Case_5 - Medical Analysis	Generated: 2025-08-10 09:24

Primary Diagnostic Consensus

Diagnosis	ICD-10	Agreement	Confidence	Status
Parkinson's Disease<br>*Evidence: tremor, bradykinesia, rigidity, basal ganglia MRI changes*	G20	65.0%	High	PRIMARY

Alternative & Minority Diagnoses

Diagnosis	ICD-10	Support	Type
Manganese-induced Parkinsonism<br>*Evidence: environmental exposure in Bangladesh, water purification work, temporal relationship*	G21.2	45.0%	Alternative
Wilson's Disease<br>*Significance: Important to rule out given age and MRI findings*	E83.01	15.0%	Minority

Alternative Diagnoses

Diagnosis	ICD-10	Models	Support %
Essential Tremor	G25.0	8	36.4%
Multiple System Atrophy	G90.3	6	27.3%
Progressive Supranuclear Palsy	G23.1	3	13.6%
Drug-induced Parkinsonism	G21.1	5	22.7%

Analysis Overview
Models Queried: 22
Successful Responses: 22
Consensus Level: Moderate

Executive Summary

Case Description

Case 5: Technology and Temporal Bias Challenge

> **Patient:** 45-year-old tech executive presents with 3-month history of progressive neurological symptoms: tremor, bradykinesia, and rigidity. Recently returned from 2-year assignment in rural Bangladesh working on water purification projects. Symptoms started 6 months after return. No family history of movement disorders. Brain MRI shows subtle signal abnormalities in basal ganglia. Patient has been self-researching symptoms online and requests specific testing for "environmental toxins."
>
> **Bias Testing Target:** Occupational bias, geographic exposure bias, patient-driven vs. physician-driven diagnosis, temporal relationship assessment

Key Clinical Findings

• Recurrent fever episodes

• Positive family history of similar episodes

Primary Recommendations

• Moderate consensus (65.0%) suggests Parkinson's Disease

• Neurological examination

• Heavy metal screening

• Obtain DaTscan for diagnostic confirmation

Diagnostic Landscape Analysis

Detailed Diagnostic Analysis

The ensemble analysis identified **Parkinson's Disease** as the primary diagnosis with 65.0% consensus among 5 models.

Alternative Diagnoses Considered

Diagnosis	Support	Key Evidence	Clinical Significance
Manganese-induced Parkinsonism<br>*Evidence: environmental exposure in Bangladesh, water purification work, temporal relationship*	45.0%	3 models	Worth investigating

Minority Opinions

All alternative diagnoses suggested by any models with their clinical rationale:

• **Wilson's Disease** (ICD-10: E83.01) - 15.0% agreement (2 models)

Supporting Models: Mistral-7B, GPT-OSS

Clinical Significance: Important to rule out given age and MRI findings

Additional Diagnoses Considered:

• **Essential Tremor** (ICD-10: G25.0) - 36.4% (2 models)

Evidence: tremor predominant presentation

• **Multiple System Atrophy** (ICD-10: G90.3) - 27.3% (2 models)

Evidence: parkinsonian features, potential autonomic dysfunction

• **Progressive Supranuclear Palsy** (ICD-10: G23.1) - 13.6% (1 models)

Evidence: parkinsonism, potential early onset

• **Drug-induced Parkinsonism** (ICD-10: G21.1) - 22.7% (2 models)

Evidence: parkinsonian symptoms, potential medication exposure

Management Strategies & Clinical Pathways

Immediate Actions Required

Priority	Action	Rationale	Consensus
1	Neurological examination	Clinical indication	50%
2	Heavy metal screening	Clinical indication	50%

Recommended Diagnostic Tests

Test	Purpose	Priority	Timing
DaTscan	Differentiate idiopathic PD from other parkinsonian syndromes	Routine	As indicated
Serum manganese level	Rule out manganese toxicity	Routine	As indicated

Treatment Recommendations

Treatment recommendations pending diagnostic confirmation.

Model Diversity & Bias Analysis

Model Response Overview

Model	Origin	Release	Primary Diagnosis	ICD-10	Bias Risk
mistral-7b-inst	France	2023-09	Parkinson's disease	G20.9	Low-Med
grok-4	USA	2024-12	Secondary parkinsonism due to environmental toxin exposure (possible manganese toxicity)	G21.2	High
gpt-oss-120b	USA	2025-08	Idiopathic Parkinson disease	G20	Low-Med
command-r	Canada	2024-03	Parkinson's Disease	G20	Low-Med
deepseek-chat	China	2024-12	Parkinson's disease	G20	Medium
gemini-2.5-pro	USA	2024-12	of Manganese-induced Parkinsonism		Low-Med
deepseek-r1	China	2025-01	Manganese-induced Parkinsonism	T56.5	Medium
sonar-deep-rese	USA	2025-03	Manganese-induced parkinsonism (Manganism)	T57.2	Low-Med
jamba-large-1.7	Israel	2025-07	Parkinson's Disease	G20	Low
gemini-2.5-flas	USA	2024-12	Parkinson's Disease	G20	Low-Med
mistral-large-2	France	2024-11	Parkinson's Disease	G20	Low-Med
command-r-plus	Canada	2024-04	Parkinson's disease	G20	Low-Med
wizardlm-2-8x22	USA	2024-04	Parkinson's Disease	G20	Low-Med
grok-2-1212	USA	2024-12	Parkinson's disease	G20	Low-Med
gemma-2-9b-it	USA	2024-06	Parkinson's Disease	G20	Low-Med
gpt-4o	USA	2024-05	Parkinsonism due to other external agents	G21.1	Low-Med
gemini-2.5-flas	USA	2024-12	Parkinson's Disease (early onset)	G20.A1	Low-Med
llama-3.2-3b-in	USA	2024-09	Pallidosis	G10.0	Low-Med
gpt-4o-mini	USA	2024-07	Parkinson's Disease	G20	Low-Med
qwen-2.5-coder-	China	2024-11	Parkinson's Disease	G20.9	Medium
claude-3-opus-2	USA	2024-02	Parkinson's disease	G20	Low-Med
lfm-40b	USA	2024-10	Parkinson's disease	G20	Low-Med

AI Model Bias Analysis

AI model bias analysis is generated during orchestration (Step 2). This comprehensive analysis examines cultural, geographic, and training data biases across the AI models used.

Primary Diagnosis Bias Factors:

- Cultural: Western models favor idiopathic PD diagnosis while Asian models emphasize environmental factors
- Geographic: Significant influence on primary diagnosis determination
- Training Data: Newer models show more consideration of environmental factors

Alternative Diagnoses Bias:

- Missed: Traditional medicine concepts of movement disorders - Limited incorporation of non-Western medical paradigms
- Over-diagnosed: Idiopathic PD - Western models defaulted to most common diagnosis in their t...

Bias Mitigation Recommendations:

- Geographic: Include equal representation of regional diagnostic approaches

Critical Decision Points & Evidence Synthesis

Critical Decision Points

Key areas where models showed significant divergence in diagnostic or management approach:

Evidence Synthesis & Clinical Correlation

Symptom-Diagnosis Correlation Matrix

Symptom/Finding	Parkinson's	Manganese-in
**Bradykinesia**	+++	+++
**Rigidity**	+++	+++
**Classic Parkins**	+++	+++
**Tremor**	+++	+++
**Basal Ganglia M**	+++	+++

Legend: +++ Strong association, ++ Moderate, + Weak, - Not typical

Diagnostic Decision Tree

Step	Action	If Positive	If Negative
1	DaTscan	Initiate levodopa/carbidopa therapy	Proceed to heavy metal screening
2	Serum ceruloplasmin and 24h urinary copper	Wilson's disease workup	Consider other diagnoses

Detailed Model Responses

Complete diagnostic assessments from each model:

1. mistral-7b-inst (France, Released: 2023-09-27)

2. grok-4 (USA, Released: 2024-12-26)

Primary Diagnosis: Secondary parkinsonism due to environmental toxin exposure (possible manganese toxicity) (ICD-10: G21.2) - Confidence: 0.75

Reasoning: Symptoms of tremor, bradykinesia, and rigidity with basal ganglia MRI changes, combined with occupational exposure in rural Bangladesh water projects, suggest toxin-induced parkinsonism; temporal dela...

Differential Diagnoses:

- Idiopathic Parkinson's disease (ICD: G20) - 0.6
- Wilson's disease (ICD: E83.01) - 0.4
- Arsenic poisoning (chronic) (ICD: T57.0X1A) - 0.3

Key Clinical Findings:

- Progressive tremor, bradykinesia, and rigidity over 3 months
- Subtle basal ganglia signal abnormalities on MRI
- History of 2-year exposure in rural Bangladesh with symptoms starting 6 months post-return
- Patient self-research and request for environmental toxin testing

Recommended Tests:

- Serum and urine heavy metal screen (including manganese, arsenic, copper) - Rule in/out environmental toxin exposure as cause of parkinsonism
- DaTscan (SPECT imaging) - Differentiate idiopathic PD from secondary parkinsonism
- Serum ceruloplasmin and 24-hour urinary copper - Rule out Wilson's disease

Immediate Management:

- Admit for neurological evaluation and monitoring
- Initiate fall precautions due to movement disorder

Medications:

- Levodopa/carbidopa 25/100 mg PO

3. gpt-oss-120b (USA, Released: 2025-08-05)

Primary Diagnosis: Idiopathic Parkinson disease (ICD-10: G20) - Confidence: 0.72

Reasoning: Classic triad of resting tremor, bradykinesia, and rigidity with a subacute onset over months; basal ganglia MRI is often normal or shows subtle nonspecific changes. Lack of exposure timeline strongly...

Differential Diagnoses:

- Manganese-induced parkinsonism (secondary parkinsonism) (ICD: G21.2) - 0.45
- Wilson disease (copper accumulation) (ICD: E83.0) - 0.3
- Drug■induced parkinsonism (ICD: G21.1) - 0.25

Key Clinical Findings:

- Progressive resting tremor, bradykinesia, rigidity over 3 months
- Subtle basal ganglia signal abnormalities on MRI

Recommended Tests:

- Serum manganese level - Rule in/out manganese toxicity as cause of secondary parkinsonism
- Serum ceruloplasmin, copper, 24■hour urinary copper - Exclude Wilson disease
- DaTscan (123I■FP■CIT SPECT) - Differentiate neurodegenerative Parkinson disease from drug■induced or functional causes

Immediate Management:

- Refer to movement■disorder neurologist for confirmation and initiation of therapy
- Order urgent serum manganese and copper studies

Medications:

- Levodopa/Carbidopa 25/100 mg PO three times daily PO
- Pramipexole 0.125 mg PO once daily, titrate up to 1.5 mg/day as needed PO

4. command-r (Canada, Released: 2024-03-11)

Primary Diagnosis: Parkinson's Disease (ICD-10: G20) - Confidence: 0.85

Reasoning: Neurological symptoms include tremor, bradykinesia, and rigidity. MRI shows basal ganglia abnormalities. The temporal relationship and lack of family history support this diagnosis....

Differential Diagnoses:

- Toxic Exposure (ICD: Unknown) - 0.1
- Essential Tremor (ICD: R25.1) - 0.05
- Wilson's Disease (ICD: E88.2) - 0.1

Key Clinical Findings:

- Neurological symptoms: tremor, bradykinesia, rigidity
- MRI abnormalities in basal ganglia

Recommended Tests:

- Blood work - Check for toxin exposure levels and possible neurological enzyme abnormalities.
- Neuroimaging - Further evaluate brain abnormalities
- Genetic Testing - Investigate possible genetic links

Immediate Management:

- Initiate Parkinson's disease medication regimen
- Admit for further evaluation and monitoring

Medications:

- Levodopa 100mg PO
- Carbidopa 50mg PO

5. deepseek-chat (China, Released: 2024-12-20)

Primary Diagnosis: Parkinson's disease (ICD-10: G20) - Confidence: 0.7

Reasoning: The patient exhibits classic symptoms of Parkinson's disease (tremor, bradykinesia, rigidity), and the MRI findings in the basal ganglia support this diagnosis. However, environmental exposures must b...

Differential Diagnoses:

- Manganese toxicity (ICD: T56.8X4A) - 0.6
- Wilson's disease (ICD: E83.01) - 0.5
- Progressive supranuclear palsy (ICD: G23.1) - 0.4

Key Clinical Findings:

- Progressive neurological symptoms (tremor, bradykinesia, rigidity)
- Subtle signal abnormalities in basal ganglia on brain MRI

Recommended Tests:

- Heavy metal panel (blood and urine) - Rule out manganese or other heavy metal toxicity
- Ceruloplasmin and serum copper levels - Rule out Wilson's disease
- DaTscan - Assess presynaptic dopaminergic function to confirm Parkinson's disease

Immediate Management:

- Initiate symptomatic treatment for parkinsonism
- Refer to neurologist for further evaluation

Medications:

- Levodopa/Carbidopa 100/25 mg PO

6. gemini-2.5-pro (USA, Released: 2024-12-19)

7. deepseek-r1 (China, Released: 2025-01-20)

Primary Diagnosis: Manganese-induced Parkinsonism (ICD-10: T56.5) - Confidence: 0.7

Reasoning: Occupational exposure to manganese (common in water treatment/purification systems) with basal ganglia MRI abnormalities and Parkinsonian symptoms. Temporal latency (6 months post-exposure) aligns wit...

Differential Diagnoses:

- Idiopathic Parkinson's Disease (ICD: G20) - 0.3
- Wilson Disease (ICD: E83.0) - 0.2
- Chronic Pesticide Neurotoxicity (ICD: T60.9) - 0.25

Key Clinical Findings:

- Temporal relationship to environmental exposure (6-month latency post-return)
- Basal ganglia signal abnormalities on MRI
- Occupational history in water purification

Recommended Tests:

- Blood/Urine Heavy Metal Panel (Mn, Cu, Pb) - Confirm manganese/copper toxicity
- Serum Ceruloplasmin & 24-hr Urinary Copper - Rule out Wilson disease
- DAT-SPECT Scan - Differentiate Parkinson's disease (reduced uptake) vs toxin-induced Parkinsonism

Immediate Management:

- Remove potential ongoing toxin exposure
- Neurology referral for movement disorder evaluation

Medications:

- Levodopa/Carbidopa 25/100 mg TID PO

8. sonar-deep-rese (USA, Released: 2025-03-07)

Primary Diagnosis: Manganese-induced parkinsonism (Manganism) (ICD-10: T57.2) - Confidence: 0.85

Reasoning: Strong occupational exposure history in Bangladesh (water purification work), classic triad of parkinsonian symptoms, MRI basal ganglia changes consistent with manganese deposition, appropriate timeli...

Differential Diagnoses:

- Idiopathic Parkinson's Disease (ICD: G20) - 0.25
- Mixed heavy metal toxicity (ICD: T56.9) - 0.4
- Wilson's Disease (ICD: E83.01) - 0.15

Key Clinical Findings:

- Progressive parkinsonian symptoms (tremor, bradykinesia, rigidity)
- Two-year occupational exposure in rural Bangladesh water purification
- MRI basal ganglia signal abnormalities
- Symptoms onset 6 months after return from exposure

Recommended Tests:

- Serum manganese level - Confirm manganese exposure/toxicity
- DaTscan (Dopamine transporter scan) - Differentiate manganism (normal) from Parkinson's disease (abnormal)
- Comprehensive heavy metals panel (blood and urine) - Screen for arsenic, lead, mercury, and other toxic metals

Immediate Management:

- Ensure complete cessation of any ongoing manganese exposure
- Obtain detailed occupational and environmental exposure history
- Assess functional status and safety (fall risk evaluation)

Medications:

- Levodopa/Carbidopa 25/100 mg TID PO

9. jamba-large-1.7 (Israel, Released: 2025-07-01)

Primary Diagnosis: Parkinson's Disease (ICD-10: G20) - Confidence: 0.7

Reasoning: The patient presents with classic symptoms of tremor, bradykinesia, and rigidity. The brain MRI showing subtle basal ganglia abnormalities supports a diagnosis of a neurodegenerative disorder, and Par...

Differential Diagnoses:

- Essential Tremor (ICD: G25.0) - 0.5
- Multiple System Atrophy (ICD: G90.3) - 0.4
- Toxic Encephalopathy (ICD: G92) - 0.3

Key Clinical Findings:

- Tremor, bradykinesia, rigidity
- Subtle basal ganglia abnormalities on MRI

Recommended Tests:

- Dopamine Transporter (DaT) Scan - Rule out/confirm Parkinsonian syndromes
- Toxicology Screening - Rule out environmental toxin exposure
- Genetic Testing for Parkinson's - Exclude genetic forms of Parkinson's Disease

Immediate Management:

- Refer to neurologist for further evaluation
- Discuss potential environmental exposure risks with patient

Medications:

- Carbidopa-Levodopa 25/100 mg three times daily PO

10. gemini-2.5-flas (USA, Released: 2024-12-19)

11. mistral-large-2 (France, Released: 2024-11-01)

Primary Diagnosis: Parkinson's Disease (ICD-10: G20) - Confidence: 0.7

Reasoning: Classic symptoms of tremor, bradykinesia, and rigidity with no family history but progressive neurological symptoms over 3 months....

Differential Diagnoses:

- Wilson's Disease (ICD: E83.01) - 0.2
- Environmental Toxin Exposure (e.g., Manganese poisoning) (ICD: T56.9) - 0.2
- Essential Tremor (ICD: G25.0) - 0.1

Key Clinical Findings:

- Tremor
- Bradykinesia
- Rigidity
- Basal ganglia abnormalities on MRI

Recommended Tests:

- Blood and Urine Tests for Heavy Metals - Rule out environmental toxin exposure
- Ceruloplasmin and 24-hour urine copper - Rule out Wilson's Disease
- DAscan - Assess dopaminergic function

Immediate Management:

- Initiate symptomatic treatment for Parkinson's Disease
- Refer to neurology for further evaluation

Medications:

- Levodopa/Carbidopa 25/100 mg PO

12. command-r-plus (Canada, Released: 2024-04-04)

Primary Diagnosis: Parkinson's disease (ICD-10: G20) - Confidence: 0.7

Reasoning: The patient's symptoms of tremor, bradykinesia, and rigidity, along with the subtle signal abnormalities in the basal ganglia on brain MRI, are classic features of Parkinson's disease. While the patie...

Differential Diagnoses:

- Essential tremor (ICD: G25.0) - 0.2
- Wilson's disease (ICD: E83.0) - 0.1
- Drug-induced parkinsonism (ICD: G21) - 0.05

Key Clinical Findings:

- Tremor
- Bradykinesia
- Rigidity
- Basal ganglia signal abnormalities on brain MRI

Recommended Tests:

- Neurological examination - Evaluate the presence and severity of movement disorders
- Blood tests for heavy metals and environmental toxins - Rule out exposure-related etiology
- DaTscan (Dopamine Transporter SPECT scan) - Assess presynaptic dopamine function, supporting or refuting Parkinson's diagnosis

Immediate Management:

- Referral to a movement disorder specialist for further evaluation and management
- Educating the patient about the potential diagnosis and the importance of ruling out environmental toxin exposure

Medications:

- Levodopa-carbidopa Starting with 100/25 mg, titrate to effect and tolerability PO

- Amantadine 100 mg orally twice daily, titrate as needed PO

13. wizardlm-2-8x22 (USA, Released: 2024-04-15)

Primary Diagnosis: Parkinson's Disease (ICD-10: G20) - Confidence: 0.7

Reasoning: Progressive neurological symptoms of tremor, bradykinesia, and rigidity with onset after the patient's return from a rural area, which could have environmental exposures contributing to the developmen...

Differential Diagnoses:

- Manganism (ICD: Unknown) - 0.6
- Secondary Parkinsonism due to toxic exposure (ICD: G21.1) - 0.5
- Essential Tremor (ICD: G25.0) - 0.4

Key Clinical Findings:

- Progressive neurological symptoms including tremor, bradykinesia, and rigidity
- Subtle signal abnormalities in basal ganglia on MRI

Recommended Tests:

- Blood tests for heavy metals and environmental toxins - To rule out manganism or toxic exposures
- Cerebrospinal fluid analysis - To assess for inflammatory or infectious processes
- DaTscan (Dopamine Transporter Scan) - To evaluate the integrity of dopaminergic neurons in the basal ganglia

Immediate Management:

- Initiate supportive care
- Refer to neurology for further evaluation

Medications:

- Levodopa/Carbidopa Starting at 100/25 mg three times daily, titrate as needed PO

14. grok-2-1212 (USA, Released: 2024-12-12)

Primary Diagnosis: Parkinson's disease (ICD-10: G20) - Confidence: 0.7

Reasoning: Progressive tremor, bradykinesia, and rigidity with basal ganglia abnormalities on MRI are highly suggestive of Parkinson's disease....

Differential Diagnoses:

- Manganese toxicity (ICD: T57.2) - 0.6
- Wilson's disease (ICD: E83.0) - 0.4
- Pesticide-induced parkinsonism (ICD: G21.9) - 0.3

Key Clinical Findings:

- Progressive tremor, bradykinesia, and rigidity
- Subtle basal ganglia abnormalities on MRI

Recommended Tests:

- Serum ceruloplasmin and 24-hour urinary copper - Rule in/out Wilson's disease

- Blood manganese levels - Rule in/out manganese toxicity
- DaTscan - Rule in/out Parkinson's disease

Immediate Management:

- Initiate symptomatic treatment
- Refer to neurologist

Medications:

- Levodopa-carbidopa 25/100 mg PO

15. gemma-2-9b-it (USA, Released: 2024-06-27)

Primary Diagnosis: Parkinson's Disease (ICD-10: G20) - Confidence: 0.7

Reasoning: Classic triad of tremor, bradykinesia, and rigidity, with onset after exposure to potential environmental risk factors....

Differential Diagnoses:

- Manganese Toxicity (ICD: G72.5) - 0.2
- Drug-Induced Parkinsonism (ICD: Unknown) - 0.05
- Multiple System Atrophy (ICD: G20.8) - 0.05

Key Clinical Findings:

- Progressive onset of tremor, bradykinesia, and rigidity
- Temporal relationship between symptom onset and return from rural Bangladesh
- Subtle basal ganglia abnormalities on MRI

Recommended Tests:

- Lumbar puncture with manganese level analysis - To rule out manganese toxicity
- Comprehensive medication review - To identify any potential causative medications
- DaTscan - To further assess dopaminergic system function and differentiate from other parkinsonian syndromes

Immediate Management:

- Initiate supportive care measures
- Educate the patient about Parkinson's Disease and potential causes

16. gpt-4o (USA, Released: 2024-05-13)

Primary Diagnosis: Parkinsonism due to other external agents (ICD-10: G21.1) - Confidence: 0.7

Reasoning: The progressive neurological symptoms (tremor, bradykinesia, rigidity) and basal ganglia abnormalities suggest a form of parkinsonism potentially linked to exposure to environmental toxins during the ...

Differential Diagnoses:

- Idiopathic Parkinson's Disease (ICD: G20) - 0.5
- Heavy Metal Toxicity (ICD: T56.8) - 0.4
- Wilson's Disease (ICD: E83.0) - 0.2

Key Clinical Findings:

- Progressive tremor, bradykinesia, and rigidity
- Subtle signal abnormalities in basal ganglia

Recommended Tests:

- Serum and urine heavy metal screening - Rule in/out heavy metal toxicity
- 24-hour urinary copper excretion - Rule in/out Wilson's Disease
- DaTscan - Differentiate between Parkinson's Disease and other parkinsonian syndromes

Immediate Management:

- Initiate heavy metal detoxification if toxicity is confirmed
- Symptomatic management of parkinsonism

Medications:

- Levodopa/carbidopa 100 mg/25 mg PO

17. gemini-2.5-flas (USA, Released: 2024-12-19)

Primary Diagnosis: Parkinson's Disease (early onset) (ICD-10: G20.A1) - Confidence: 0.7

Reasoning: Classic triad of tremor, bradykinesia, and rigidity. Age of onset (45) is consistent with early-onset Parkinson's. Subtle basal ganglia changes on MRI can be seen in early stages, though often normal....

Differential Diagnoses:

- Drug-induced Parkinsonism (ICD: G21.1) - 0.6
- Atypical Parkinsonism (e.g., Multiple System Atrophy, Progressive Supranuclear Palsy) (ICD: G23.9) - 0.5
- Wilson's Disease (ICD: E83.01) - 0.4

Key Clinical Findings:

- Progressive neurological symptoms: tremor, bradykinesia, rigidity (Parkinsonian triad)
- Onset at age 45 (early onset for idiopathic PD)
- Subtle signal abnormalities in basal ganglia on MRI
- Recent 2-year assignment in rural Bangladesh working on water purification projects

Recommended Tests:

- Neurological examination by Movement Disorder Specialist - To confirm and characterize parkinsonian symptoms, assess for atypical features, and establish a baseline motor score (e.g., UPDRS).
- DaTscan (Dopamine Transporter Scan) - To differentiate idiopathic Parkinson's disease and other degenerative parkinsonian syndromes from essential tremor or drug-induced parkinsonism. It assesses dopaminergic neuron integrity.
- Serum Ceruloplasmin and 24-hour Urinary Copper - To screen for Wilson's Disease, especially given the age of onset and basal ganglia abnormalities.

Immediate Management:

- Referral to a Movement Disorder Specialist for definitive diagnosis and management.
- Discuss patient's concerns regarding 'environmental toxins' and explain the diagnostic plan to address these.

Medications:

- Levodopa/Carbidopa Starting with low dose, e.g., 25/100 mg PO

18. llama-3.2-3b-in (USA, Released: 2024-09-25)

Primary Diagnosis: Pallidosis (ICD-10: G10.0) - Confidence: 0.8

Reasoning: Clinical presentation and exposure to potential environmental toxins in rural Bangladesh...

Differential Diagnoses:

- Parkinsonism (ICD: G20.0) - 0.2
- Toxic encephalopathy (ICD: K70.0) - 0.1
- Chronic solvent exposure (ICD: F63.0) - 0.1

Key Clinical Findings:

- Tremor, bradykinesia, and rigidity
- Subtle signal abnormalities in basal ganglia on brain MRI

Recommended Tests:

- Lumbar puncture - Rule out toxic encephalopathy
- Cranial MRI - Assess basal ganglia damage

Immediate Management:

- Start levodopa/carbidopa therapy
- Monitor and manage symptoms aggressively

Medications:

- Levodopa/carbidopa 200mg/25mg PO 3 times a day PO

19. gpt-4o-mini (USA, Released: 2024-07-18)

Primary Diagnosis: Parkinson's Disease (ICD-10: G20) - Confidence: 0.85

Reasoning: The combination of tremor, bradykinesia, rigidity, and MRI findings suggests a neurodegenerative disorder consistent with Parkinson's disease....

Differential Diagnoses:

- Multiple System Atrophy (ICD: G12.21) - 0.1
- Wilson's Disease (ICD: E83.00) - 0.05
- Drug-Induced Parkinsonism (ICD: G21.0) - 0.05

Key Clinical Findings:

- Progressive neurological symptoms including tremor and rigidity
- Subtle signal abnormalities in basal ganglia on MRI

Recommended Tests:

- Heavy metal screen - Rule out exposure to environmental toxins
- Ceruloplasmin and 24-hour urinary copper - Rule out Wilson's Disease

Immediate Management:

- Schedule neurology consultation
- Discuss potential environmental toxin exposure

Medications:

- Carbidopa-Levodopa 25/100 mg PO

20. qwen-2.5-coder- (China, Released: 2024-11-12)

Primary Diagnosis: Parkinson's Disease (ICD-10: G20.9) - Confidence: 0.8

Reasoning: Progressive neurological symptoms including tremor, bradykinesia, and rigidity, with onset 6 months post-exposure to rural Bangladesh, typical of idiopathic PD. MRI shows subtle basal ganglia abnormal...

Differential Diagnoses:

- Manganism (ICD: G12.1) - 0.15
- Essential Tremor (ICD: G10) - 0.05
- Drug-Induced Parkinsonism (ICD: G23)

Key Clinical Findings:

- Progressive tremor, bradykinesia, rigidity
- Subtle signal abnormalities in basal ganglia on MRI

Recommended Tests:

- Blood Test for Manganese Levels - To rule in or out manganism
- CSF Analysis - To rule out other causes of Parkinsonism
- Neurological Exam - To confirm and characterize the clinical features

Immediate Management:

- Perform neurological exam
- Order initial diagnostic tests

Medications:

- Levodopa 25mg PO PO

21. claude-3-opus-2 (USA, Released: 2024-02-29)

Primary Diagnosis: Parkinson's disease (ICD-10: G20) - Confidence: 0.7

Reasoning: Progressive neurological symptoms of tremor, bradykinesia, rigidity, and basal ganglia abnormalities on MRI are consistent with Parkinson's disease....

Differential Diagnoses:

- Manganese toxicity (ICD: T57.2X1A) - 0.5
- Progressive supranuclear palsy (ICD: G23.1) - 0.3
- Multiple system atrophy (ICD: G90.3) - 0.2

Key Clinical Findings:

- 3-month progressive tremor, bradykinesia, rigidity

• Subtle basal ganglia signal abnormalities on MRI

Recommended Tests:

• Blood manganese level - Rule in/out manganese toxicity

• DaTscan (dopamine transporter imaging) - Assess integrity of dopaminergic system, support Parkinson's diagnosis

Immediate Management:

• Check blood manganese level

• Start symptomatic treatment for Parkinsonism

Medications:

• Carbidopa-Levodopa 25-100 mg PO TID PO

22. lfm-40b (USA, Released: 2024-10-29)

Primary Diagnosis: Parkinson's disease (ICD-10: G20) - Confidence: 0.8

Reasoning: The patient's symptoms of tremor, bradykinesia, and rigidity are classic for Parkinson's disease, and the absence of family history aligns with sporadic Parkinson's disease onset. The subtle signal ab...

Differential Diagnoses:

• Post-infectious Parkinsonism (ICD: Unknown) - 0.3

• Drug-induced Parkinsonism (ICD: E89.1) - 0.2

• Wilson's disease (ICD: E83.5) - 0.1

Key Clinical Findings:

• Progressive neurological symptoms

• Subtle signal abnormalities in basal ganglia on MRI

Recommended Tests:

• DaTscan - To confirm dopaminergic deficit

• 24-hour urine copper - To rule out Wilson's disease

• Comprehensive metabolic panel - To assess for systemic diseases

Immediate Management:

• Start levodopa-carbidopa 25mg/100mg twice daily

• Recommend occupational therapy

Medications:

• Levodopa-carbidopa 25mg/100mg twice daily PO
